# Retraction: A novel concept and design for highly efficient photoelectrocatalytic materials with high performance, stability, and charge transport properties: development of an innovative next-generation green technology

**DOI:** 10.1039/d4ra90123a

**Published:** 2024-10-14

**Authors:** Abdul Qayoom Mugheri, Kashif Ali, Ali Asghar Sangah, Mazhar Iqbal Khaskheli, Muhammad Younis Laghari, Nadeem Ahmed Mugheri, Muhammad Rajib Soomro, Muhammad Ishfaq Chohan, Arsalan Ahmed Mugheri, Aftab Kandhro

**Affiliations:** a Dr M. A Kazi Institute of Chemistry, University of Sindh Jamshoro 76080 Sindh Pakistan a.qmugheri87@gmail.com; b Hunan Key Laboratory for Super Microstructure and Ultrafast Process, School of Physics and Electronics, Central South University Changsha 410083 China; c Department of Basic Sciences and Related Studies, Mehran University of Engineering and Technology Jamshoro 76080 Pakistan; d Department of Chemistry, GC University Hyderabad Pakistan; e The Department of Fisheries and Aquatic Sciences, University of Sindh Jamshoro 76080 Sindh Pakistan

## Abstract

Retraction of “A novel concept and design for highly efficient photoelectrocatalytic materials with high performance, stability, and charge transport properties: development of an innovative next-generation green technology” by Abdul Qayoom Mugheri *et al.*, *RSC Adv.*, 2024, **14**, 1581–1592, https://doi.org/10.1039/D3RA05126A.

The Royal Society of Chemistry hereby wholly retracts this *RSC Advances* article due to concerns with the authenticity of the data.

Concerns were raised that the data in the figures had been taken from another paper,^1^ the text had been re-written slightly and the names of the materials employed were changed. The authors provided raw data, but an independent expert concluded that it is not genuine.

Given the significance of these concerns, the findings presented in this paper are no longer reliable.

The authors do not agree with the decision to retract the article.

Signed: Laura Fisher, Executive Editor, *RSC Advances*

Date: 2nd Oct 2024
